# Assembled Carbon Nanostructure Prepared by Spray Freeze Drying for Si-Based Anodes

**DOI:** 10.3390/nano15090661

**Published:** 2025-04-26

**Authors:** Wanxiong Zhu, Liewen Guo, Kairan Li, Mengxue Shen, Chang Lu, Zipeng Jiang, Huaihe Song, Ang Li

**Affiliations:** 1 State Key Laboratory of Chemical Resources Engineering, Beijing Key Laboratory of Electrochemical Process and Technology for Materials, Beijing University of Chemical Technology, Beijing 100029, China; 2 Qinghai Provincial Key Laboratory of Advanced Materials and Applied Technology, Qinghai University, Xining 810016, China; 3 College of Chemical Engineering, Qinghai University, Xining 810016, China

**Keywords:** silicon anode, spray freeze drying, carbon nanostructures, polymer self-assembly, lithium-ion battery

## Abstract

Silicon-based materials provide a new pathway to break through the energy storage limits of battery systems but their industrialization process is still constrained by inherent diffusion hysteresis and unstable electrode structures. In this work, we propose a novel structural design strategy employing a modified spray freeze drying technique to construct multidimensional carbon nanostructures. The continuous morphological transition from carbon nanowires to carbon nanosheets was facilitated by the inducement of ultralow-temperature phase separation and the effect of polymer self-assembly. The unique wrinkled carbon nanosheet encapsulation effectively mitigated the stress concentration induced by the aggregation of silicon nanoparticles, while the open two-dimensional structure buffered the volume changes of silicon. As expected, the SSC-5M composite retained a reversible capacity of 1279 mAh g^−1^ after 100 cycles at 0.2 C (1 C = 1700 mAh g^−1^) and exhibited a capacity retention of 677.1 mAh g^−1^ after 400 cycles at 1 C, demonstrating excellent cycling stability. This study offers a new strategy for the development of silicon-based energy storage devices.

## 1. Introduction

In the context of global climate change mitigation, electric vehicles (EVs) have been regarded as an important development direction to achieve carbon emission reduction in the transport sector. However, the insufficient driving range remains a key factor restricting the large-scale promotion and application of EVs [1,2]. Therefore, enhancing the energy density of electrode materials has emerged as a paramount challenge, driving innovation in the field of materials. The exceptional lithium storage capability of silicon (Si) endows it with a specific capacity reaching 3579 mAh g^−1^ [3], which establishes Si-based anodes as promising alternatives to commercial carbon materials in lithium-ion batteries (LIBs). However, the substantial volume changes during the cycling process of Si destabilize the initially formed solid electrolyte interphase (SEI) and cause fractures in the active material [4,5]. As a result, these effects ultimately lead to capacity degradation of the battery. In addition, as a typical semiconductor, Si inherently suffers from insufficient electrical conductivity [6].

In general, smaller Si particles can effectively alleviate the stress resulting from volume changes during cycling. According to previous reports, when the Si particle diameter falls below a critical threshold of approximately 150 nm, the stress generated by lithiation is insufficient to cause particle rupture [7]. Therefore, the structural design of Si into low-dimensional and nanoscale structures, such as silicon nanoparticles (Si NPs), porous Si, Si sheets, and Si nanowires [8,9,10,11], can not only form a stable SEI film but also alleviate the mechanical stress induced by volume changes. However, nanoscale Si-based materials are prone to aggregation due to the high specific surface energy, posing challenges in electrode fabrication. The inherent low electrical conductivity of Si-based nanomaterials limits the rapid transport of Li^+^ in anodes. Carbon materials have been widely used due to their excellent electrical conductivity, diverse structural designs, good SEI film formation ability, and superior physicochemical stability [12]. Therefore, incorporating carbon components can effectively mitigate the volumetric changes in Si and enhance its low electrical conductivity [13,14,15]. Rational structural designs, such as watermelon-like, yolk–shell, core–shell, and sandwich structures [16,17,18,19], further enhance the advantages of carbon materials, demonstrating significant improvements in reversible capacity and cycling stability. However, these structures often require complex fabrication processes, expensive raw materials, and the use of environmentally harmful reagents, severely limiting their industrial application. Therefore, developing simple and efficient structural optimization strategies has become a key focus in the research of silicon/carbon composite (Si/C) materials.

Freeze drying has become a widely used synthesis method for nanostructured carbon materials [20,21,22]. It consists of two primary processes, freezing and drying. During the freezing process, the precursor solution undergoes controlled cooling in a refrigeration system, allowing gradual solvent crystallization and complete solid matrix formation. Subsequently, the frozen solvent is removed via sublimation in vacuum conditions. However, conventional freeze drying processes exhibit inherent limitations, including a prolonged dehydration duration and inadequate morphological control over carbon structures. To address these problems, spray-assisted freezing has been adopted to control the oriented growth of ice crystals throughout the freezing process, enabling better control over the microstructure of the material. Additionally, this method reduces the drying time by approximately 60% [23], significantly enhancing process efficiency.

In this study, we developed an innovative structural design strategy utilizing a modified freeze drying technique to prepare carbon nanomaterials. The chitosan-derived carbon nanosheets uniformly encapsulated the Si NPs, effectively isolating them from direct exposure to the electrolyte. The open structure accommodated the substantial volumetric expansion of Si by providing elastic buffering space, thereby mitigating mechanical stress and preventing structural degradation. The SSC-5M composite exhibited outstanding cycling stability, retaining a reversible capacity of 1279 mAh g**^−^**^1^ after 100 cycles at 0.2 C. Notably, it maintained 677.1 mAh g**^−^**^1^, even after 400 cycles at 1 C. This approach offers new directions and insights for Si-based energy storage devices.

## 2. Materials and Methods

Materials. Si NPs with a diameter of approximately 50 nm were purchased from Hefei Kaier (Hefei, China), as well as chitosan (Macklin, Shanghai, China; deacetylation degree 90%), acetic acid (Macklin AR; >99%).

The preparation of SSC composites was as follows. Firstly, 0.2 g, 0.4 g, 1 g, and 2 g chitosan, respectively, were added to 200 mL deionized water and stirred for 10 min to achieve preliminary dispersion. Subsequently, acetic acid was added in amounts of 0.1 mL, 0.2 mL, 0.5 mL, and 1 mL according to the increasing amount of chitosan to facilitate dissolution, resulting in chitosan solutions with concentrations of 1 g·L^−1^, 2 g·L^−1^, 5 g·L^−1^, and 10 g·L^−1^. Additionally, the Si NPs were dispersed in 10 mL anhydrous ethanol at a mass ratio of chitosan to Si of 10:3 and stirred for 12 h. The Si dispersion was poured into the chitosan solution and stirred for 12 h. After mixing was completed, the mixture was sprayed into liquid nitrogen and quickly transferred to a lyophilizer for 36 h at −95 °C and 0.01 Pa. Finally, the dried sample was placed in a tube furnace. Under a high-purity argon atmosphere (100 mL min^−1^; 99.999%), it was heated to 800 °C at a rate of 5 °C min^−1^ and maintained at this temperature for 2 h to obtain the SSCs. According to the chitosan concentration, the samples were named SSC-1M, SSC-2M, SSC-5M, and SSC-10M, respectively.

Material Characterization. The morphology and encapsulation of the samples were characterized using scanning electron microscopy (SEM) (Zeiss SUPRATM 55; Oberkochen, Germany) and transmission electron microscopy (TEM, Puchong, Malaysia) (HT7700). The composition of the materials was analyzed using X-ray diffraction (XRD) (Rigaku Cu Kα, λ = 1.540 56 Å; Tokyo, Japan) and Raman spectroscopy (Aramis, Jobin Yvon, Arcueil, France; 532 nm laser light). The surface elements of the material were analyzed using X-ray photoelectron spectroscopy (XPS) (Thermo, Waltham, MA, USA; 250Xi). The Si and carbon contents of the samples were tested via a thermogravimetric analysis (TGA) (STA7300 HTIACHI, Tokyo, Japan) by heating to 800 °C under air conditions at a ramp rate of 10 °C/min.

Electrochemical Measurements. The working electrode was prepared by mixing the active material, carbon black, and carboxymethyl cellulose at a weight ratio of 8:1:1 in deionized water to form a slurry. The slurry was uniformly coated onto copper foil with a carbon substrate using a 200 μm doctor blade and then dried in a vacuum oven at 100 °C for 12 h. The dried electrode was cut into discs with diameters of 12 mm, and the active material loading was controlled at approximately 1 mg cm^−2^. Half-cells were assembled in an argon-filled glove box using lithium foil as the counter electrode, Celgard 2500 as the separator, and 1 M LiPF6 dissolved in ethylene carbonate (EC) and dimethyl carbonate (DMC) (EC:DMC mass ratio = 1:1) with 5% fluorinated ethylene carbonate (FEC) as the electrolyte. The electrochemical performance of the samples was evaluated using CR2032 coin cells. All half-cells were tested using constant-current charge/discharge over a voltage range of 0.005–1.5 V. Additionally, cyclic voltammetry (CV) and electrochemical impedance spectroscopy (EIS) tests were conducted on a CHI600E electrochemical workstation. The CV test was performed over a voltage range of 0.005–1.5 V for the first three cycles at a scanning rate of 0.1 mV s^−1^, while the EIS test was carried out in the frequency range of 100 kHz to 0.1 Hz. The full cell was assembled using a composite anode composed of SSC-5M and commercial graphite at a mass ratio of 2:8, paired with a commercial LiFePO_4_ cathode (N/P ratio of 1.1). Prior to assembly, the anode was prelithiated through direct contact with lithium foil. The electrochemical performance of the full cell was evaluated over a voltage range from 2.2 V to 4.0 V.

## 3. Results and Discussion

**Figure 1:**
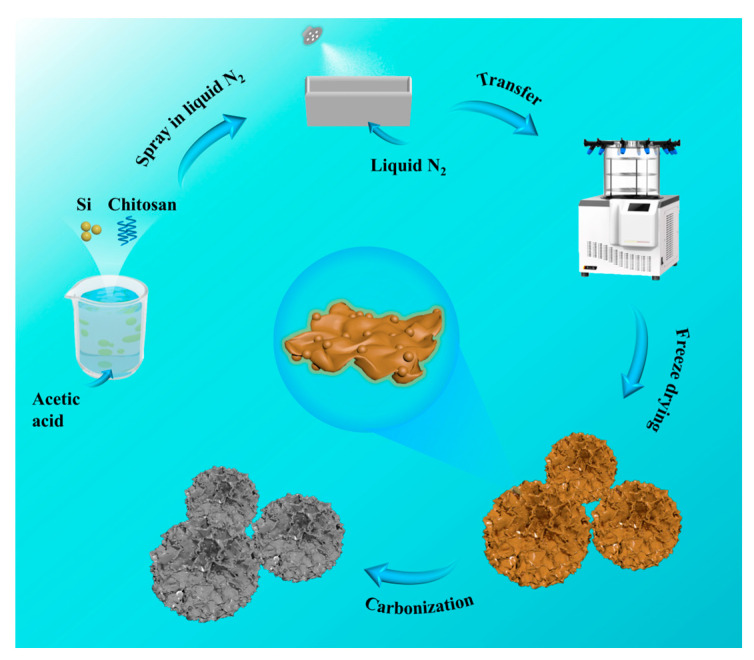
The preparation process illustration of SSC-5M.

The synthesis procedure of SSC-5M is illustrated in Figure 1. Initially, chitosan and Si were homogeneously dispersed in an acetic acid solution. Subsequently, the mixture was sprayed into liquid nitrogen. A significant temperature gradient was established from the exterior to the interior of the droplets upon contact with the liquid nitrogen. Simultaneously, the crystallization of the water at the droplet surface initiated phase separation between the water and chitosan, driving the migration of the chitosan toward the interior of the droplet [24]. Finally, ice crystals were removed via lyophilization followed by thermal treatment, resulting in the formation of spherical assembled structures with radial channels whose diameters ranged from 10 to 60 µm (Figure S1).

**Figure 2:**
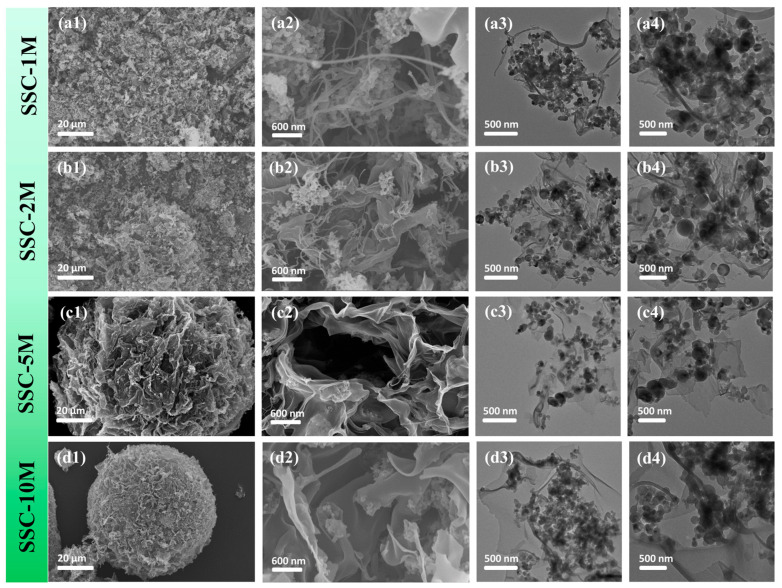
SEM images of (**a1**,**a2**) SSC-1M, (**b1**,**b2**) SSC-2M, (**c1**,**c2**) SSC-5M, and (**d1**,**d2**) SSC-10M, and TEM images of (**a3**,**a4**) SSC-1M, (**b3**,**b4**) SSC-2M, (**c3**,**c4**) SSC-5M, and (**d3**,**d4**) SSC-10M.

To investigate the formation mechanism of carbon nanostructures, a systematic comparative study was conducted on samples prepared at different chitosan concentrations. At a concentration of 1 g L^−1^, the obtained structures mainly consisted of numerous carbon nanowires and a small number of carbon nanosheets (Figure 2(a1,a2)), in which the Si nanoclusters were entangled by nanowires (Figure 2(a3,a4)). With an increase in the chitosan concentration to 2 g L^−1^, the amount of carbon nanosheets significantly increased and the dispersion of Si particles significantly improved (Figure 2(b1–b4)). When the chitosan concentration was increased to 5 g L^−1^, a spherical structure composed of folded carbon nanosheets was assembled, in which the Si NPs were uniformly and homogeneously embedded in the carbon nanosheets (Figure 2(c1–c4)). However, at a concentration of 10 g L^−1^, the spherical structure formed by the assembly of carbon nanosheets became more compact, and the size of individual carbon sheets considerably expanded but the dispersion of Si NPs started to decrease (Figure 2(d1–d4)). This suggests that controlling the chitosan concentration significantly influences the microstructure of carbon nanostructures and the dispersion of Si NPs. This structural transformation was caused by the high surface tension of the droplets generated during the spraying process. According to the Gibbs–Thomson equation (Equation (S1)) [25], the melting point of a droplet system is significantly lowered when the droplets have a positive average curvature. Therefore, polymer crystals are more susceptible to fragmentation during the solidification process, resulting in smaller polymer embryos. According to the polymer interface nucleation rate equation (Equation (S2)) [25], an increase in the polymer concentration leads to an elevation in the embryo density. Subsequently, guided by the ice template, these high-density polymer embryos undergo further fusion, facilitating the transformation of carbon nanowires into carbon nanosheet structures.

**Figure 3:**
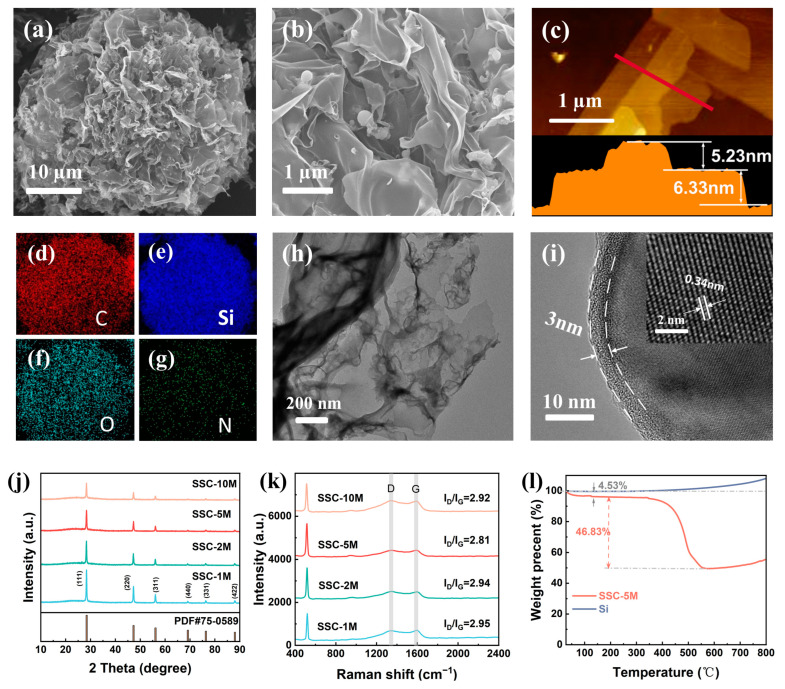
(**a**,**b**) SEM images of SSC-5M. (**c**) AFM image and height profile of SSC-5M. (**d**–**g**) Elemental distribution diagrams of SSC-5M. (**h**) TEM images of SSC-5M after the removal of Si NPs. (**i**) HRTEM and crystal magnification image of SSC-5M. (**j**) XRD patterns of SSCs. (**k**) Raman spectra of SSCs. (**l**) TGA test of SSC-5M.

The morphology of the SSC-5M composite material was analyzed using SEM and TEM. As shown in Figure S2a, the Si NPs with a particle size of approximately 50 nm tended to agglomerate due to surface energy. The chitosan spherical structures had a size of approximately 50 µm (Figure S2b). After mixing Si NPs with protonated chitosan, the chitosan molecules were easily adsorbed onto the surface of the Si NPs, forming an encapsulated structure via electrostatic attraction [26]. Figure 3a shows the morphology of SSC-5M, which exhibited a flower-ball structure composed of carbon nanosheets. These carbon nanosheets displayed a distinct wrinkled appearance and grew radially outward from the core of the sphere. The SEM magnified image (Figure 3b) clearly shows that the Si NPs were uniformly embedded within the carbon nanosheets. Furthermore, the chitosan-derived carbon nanosheets had a thickness of only 5–6 nm (Figure 3c) and their low dimensionality shortened the transport path of Li^+^. Additionally, the elemental distribution diagrams of SSC-5M showed the uniform distribution of C, Si, O, and N (Figure 3d–g), indicating that the Si NPs were evenly dispersed within the spherical assembled structures. The nucleophilic elements O and N, derived from the glucosamine units in chitosan, provide numerous active sites to facilitate the migration of Li^+^ [27]. After the removal of Si NPs, vesicle-like structures were observed to be distributed within the carbon nanosheets (Figure 3h), confirming that the chitosan-derived carbon not only formed a nanosheet network but also established an encapsulated layer on the surface of Si NPs [5]. HRTEM and high-resolution lattice images showed that the Si NPs attached to the surface were uniformly encapsulated by approximately 3 nm thick chitosan-derived pyrolyzed carbon (Figure 3i), effectively shielding them from direct exposure to the electrolyte. The lattice spacing of 0.34 nm aligned with the (111) crystal plane of Si [28]. The exceptional mechanical flexibility of the carbon nanosheets effectively mitigated the structural degradation induced by repeated volume fluctuations in the electrode materials.

Figure 3j shows the XRD patterns of SSCs. The broad characteristic peaks at 23° corresponded with the (002) planes of the amorphous carbon structure. Furthermore, the other characteristic peaks aligned with the Si PDF standard card [29]. No characteristic peaks associated with Si carbide were observed, indicating that the single-crystal Si maintained its original structure after pyrolysis. Moreover, with a similar Si content, the intensity of the Si characteristic peaks gradually decreased with an increase in chitosan concentration. This suggests that the carbon nanostructures significantly enhanced their coating of Si particles under higher chitosan concentrations. In the Raman spectra (Figure 3k), the characteristic vibrational modes at 512 cm^−1^ and 934 cm^−1^ were assigned to crystalline Si, while distinct characteristic peaks emerged at 1344 cm^−1^ (D-band) and 1588 cm^−1^ (G-band) [26], respectively. The D-band arises from the breathing mode of sp^3^-hybridized carbon atoms in disordered structures, while the G-band originates from the in-plane vibrations of sp^2^-bonded carbon pairs in graphitic domains [30]. The ratio of I_D_/I_G_ is commonly used to characterize the degree of graphitization. For sample SSC-5M, the I_D_/I_G_ value was 2.81 (Figure S3c), indicating a low degree of graphitization, likely due to the disordered hard carbon structure derived from chitosan. To determine the proportions of Si and C in the composite material, a TGA of the SSCs was conducted in an air atmosphere (Figure 3l and Figure S4). The results revealed a mass loss of approximately 4.5% from room temperature to 150 °C, primarily attributed to the evaporation of adsorbed moisture. Subsequently, the mass remained relatively stable. As the temperature increased to 400 °C, the pyrolyzed carbon derived from chitosan began to react with oxygen, leading to decomposition and the rapid release of small gaseous molecules [26], resulting in a significant mass loss of the composites. This degradation process approached completion around 550 °C. Due to the oxidation of Si, the TGA curves exhibited a gradual increase in mass beyond 550 °C. After accounting for the weight gain resulting from Si oxidation, the Si contents in SSC-1M, SSC-2M, SSC-5M, and SSC-10M were estimated to be 49.6%, 48.5%, 49.2%, and 49.3%, respectively.

The surface chemical composition of SSC-5M was analyzed using XPS. As shown in Figure S5a, SSC-5M consisted of the following four elements: C (64.54%), Si (14.89%), O (11.57%), and N (8.97%). Notably, the Si content measured using XPS was significantly lower than that determined by the TGA, primarily due to the shielding of Si signals by the overlying carbon nanosheets and layers; the detecting depth of XPS is only a few atom-layers. As shown in Figure S5b, pyrolysis promoted the formation of C-C, C-N, and C-O bonds [31,32]. Si NPs exhibit high surface activity, which allows their surface to readily form an oxide layer. As a result, the Si-O bond signal was stronger than the Si 2p_1/2_ and Si 2p_3/2_ signals (Figure S5c) [33]. The N 1s signal consisted of three peaks corresponding with pyridine nitrogen, pyrrole nitrogen, and graphite nitrogen, respectively (Figure S5d) [34], which proved that elemental N was successfully doped into the SSC-5M composites during the carbonization of chitosan. Pyridine nitrogen and pyrrole nitrogen provide additional storage sites for Li^+^, while graphite nitrogen improves the electrical conductivity of composites [35]. Nitrogen adsorption–desorption tests provide deeper insights into the pore structure of composite materials. Figure S6 shows that all samples exhibited type II isotherms [36], indicating that the pore structures of the composites primarily consisted of macropores or non-porous regions. The formation of macropores is closely associated with the sublimation of ice crystals during the freeze drying process. As shown in Table S1, the average pore diameters of SSC-1M, SSC-2M, SSC-5M, and SSC-10M were 21.29, 25.47, 17.81, and 15.94 nm, respectively, generally decreasing with an increase in chitosan concentration. The higher density of the chitosan embryos facilitated a reduction in the gaps between materials during the mutual fusion of the nanostructures. Specific surface area measurements revealed values of 46.44, 50.68, 59.68, and 72.23 m^2^/g for SSC-1M, SSC-2M, SSC-5M, and SSC-10M, respectively. These findings indicate that two-dimensional carbon nanosheets possess larger specific surface areas and smaller pore sizes than one-dimensional carbon nanowires. This structure provided more attachment sites for Si NPs, accelerating Li^+^ migration on the Si surface.

**Figure 4:**
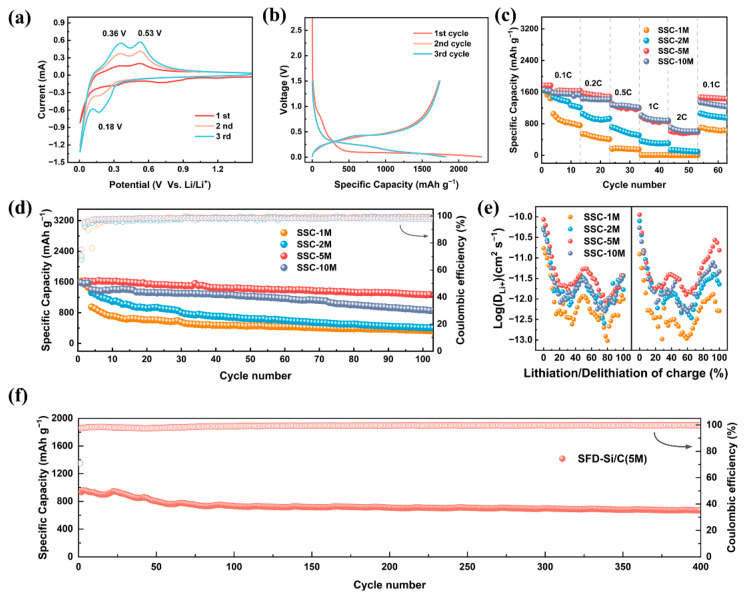
(**a**) CV profiles of SSC-5M. (**b**) GCD profiles of SSC-5M at a 50 mA g^−1^ current density. (**c**) Rate performance of SSCs. (**d**) Cycle performance of SSCs at a 0.2 C current density (1 C = 1700 mA g^−1^). (**e**) Lithium-ion diffusion coefficient diagram. (**f**) Long-term cycling performance of SSC-5M.

The CV profiles of SSC-5M were measured at a scanning rate of 0.1 mV s^−1^ over a voltage window of 0.005–1.5 V (Figure 4a). During the initial cathodic sweep, a broad reduction peak was observed between 0.5 and 1 V, corresponding with electrolyte decomposition and the formation of an SEI layer. The absence of this peak in subsequent cycles demonstrated the stability of the SEI layer established during the initial cycle [37]. Another cathodic peak was observed at approximately 0.18 V, corresponding with the alloying process of Si. During the anodic sweep, peaks at 0.36 V and 0.53 V corresponded with the oxidation-driven delithiation of Li_x_Si [38,39]. Moreover, the redox peaks gradually increased in intensity with increasing cycle numbers, suggesting that the activity of the electrode material was progressively released. The galvanostatic charge–discharge (GCD) profiles of SSC-5M exhibited a discharge plateau at 0.8 V during the initial discharge, which vanished in later cycles, demonstrating the formation of a stable SEI layer (Figure 4b) [40]. In comparison, SSC-1M and SSC-2M lacked this distinctive 0.8 V plateau (Figure S7), suggesting their inability to establish a stable SEI layer. Figure 4b shows that SSC-5M had an initial discharge capacity of 1731.06 mAh g^−1^ and a coulombic efficiency of 74.94% (Figure 4b). The low initial coulombic efficiency (ICE) was primarily attributed to two factors: the trapping of Li^+^ by defects of the poorly graphitized carbon and excessive SEI formation caused by the carbon nanostructure [41].

Figure 4c shows the rate performance test results of the SSCs. SSC-5M and SSC-10M exhibited excellent rate performance, maintaining 53.5% and 57.3% capacity retention at 1 C (in comparison to the capacity at 0.1 C), respectively. The carbon nanosheets effectively accelerated the surface reaction kinetics and enhanced the rate performance. In contrast, SSC-1M and SSC-2M, which were primarily composed of carbon nanowires, showed much lower capacity retention at 1 C, with values of only 22.3% and 0.92%, respectively. SSC-5M also demonstrated outstanding cycling stability, retaining a specific capacity of 1279 mAh g^−1^ after 100 cycles at 0.2 C. In contrast, SSC-1M, SSC-2M, and SSC-10M showed specific capacities of only 337 mAh g^−1^, 428 mAh g^−1^, and 889 mAh g^−1^ after 100 cycles (Figure 4d), respectively. Overall, SSC-5M exhibited the best overall performance due to its abundant carbon nanosheets and excellent Si dispersion. The Si/C prepared by spray freeze drying technology demonstrated enhanced electrochemical performance compared with those prepared by other methods (Table S2).

To investigate Li^+^ diffusion behavior in the electrode materials, the galvanostatic intermittent titration technique (GITT) was utilized to evaluate the composites. Assuming that Li^+^ diffusion adhered to Fick’s second law, the diffusion coefficient of Li^+^ was determined using Equation S(3) [42]. Compared with the other samples, SSC-5M demonstrated significantly higher $D_{{Li}^{+}}$ during the charge–discharge process (Figure 4e), indicating superior electrochemical dynamics and rate performance. Figure 4f illustrates the long-term cycling performance of SSC-5M at 1 C. After the pre-cycling process, the results indicated that SSC-5M retained a specific capacity of 677.1 mAh g^−1^, with a high coulombic efficiency of 99.7% after 400 cycles, demonstrating outstanding cycling stability. This advantage was due to its rational structural design. The interconnected architecture of two-dimensional carbon sheets markedly enhanced the interfacial contact between the carbon additives and electrode components [43], thereby maintaining uninterrupted electron pathways from the electrode substrate to the active material. This structural configuration substantially improved both Li^+^ migration kinetics and charge transfer efficiency across the Si-based anode.

**Figure 5:**
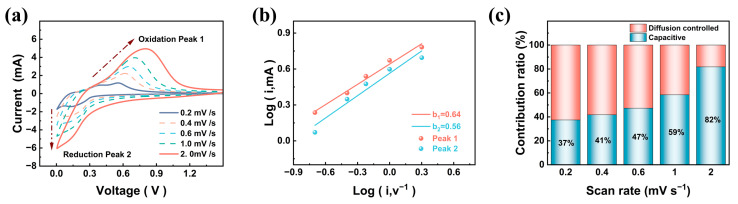
(**a**) CV curves of SSC-5M at different scan rates. (**b**) Relationship between the peak currents and scan rates of SSC-5M. (**c**) Capacitive contributions of SSC-5M at different scan rates.

The CV tests were conducted at scan rates of 0.2–2 mV s^−1^ to examine the reaction kinetics and Li^+^ storage mechanism. Figure 5a shows that all CV curves had similar shapes, indicating that the SSC-5M electrode had high reversibility and low polarization. When the scan rate increased, the redox peaks of SSC-5M became more pronounced and shifted, covering a wider voltage range. Based on the calculation of b-values for the redox peaks using Equation S(4), values of 0.59 and 0.62 were obtained, respectively (Figure 5b). This suggests that Li^+^ storage in SSC-5M involved both diffusion-controlled and capacitive processes. As shown in Figure 5c, the capacitive contribution increased with the scan rate, with SSC-5M exhibiting a 59% capacitive contribution at 1 mV s^−1^. Compared with the other SSCs (Figure S8), it was found that the carbon nanosheets could provide higher capacitance. This could be attributed to the ultrathin carbon nanosheets, providing a large surface area with abundant active sites, and their unique two-dimensional structure, which significantly shortened the Li^+^ diffusion pathways [44].

**Figure 6:**
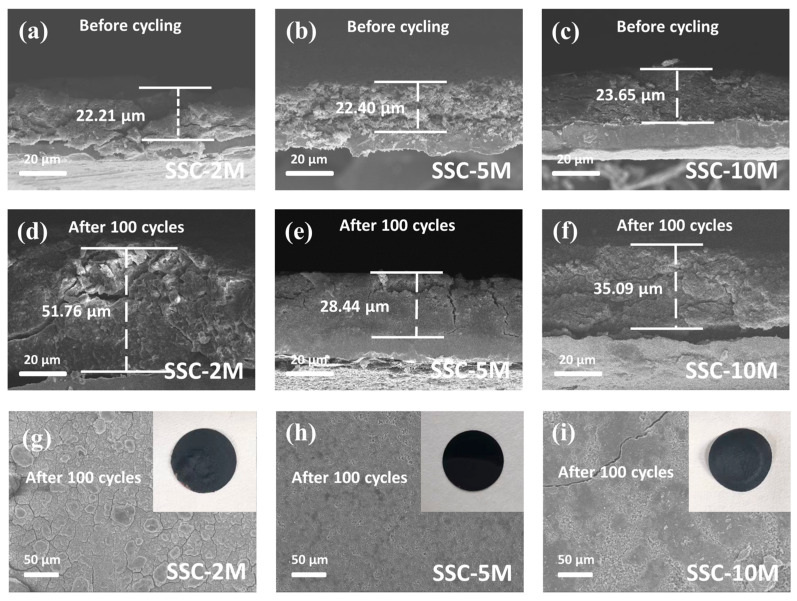
Cross-sectional SEM images of SSC-2M, SSC-5M, and SSC-10M electrodes (**a**–**c**) before cycling and (**d**–**f**) after 100 cycles. (**g**–**i**) Surface morphology SEM images of SSC-2M, SSC-5M, and SSC-10M electrodes after 100 cycles.

Different types of carbon nanostructures exhibited varying effects in stabilizing the electrode structure. The results showed that the initial thickness of the SSC-2M electrode was 22.21 µm, which increased to 51.67 µm after 100 cycles (Figure 6a,d), corresponding with a growth ratio of 132.67%. Such excessive electrode expansion can lead to the rupture of the SEI layer and the detachment of the active material, ultimately causing rapid capacity degradation. In contrast, electrodes with abundant two-dimensional structures exhibit significantly lower volume changes. The thickness of the SSC-5M and SSC-10M electrodes increased to 28.44 µm and 35.0 µm (Figure 6e,f), with growth rates of only 26.96% and 48.37%, respectively, substantially lower than the SSC-2M electrode. The surface morphology of the electrodes provided additional evidence for this conclusion. After 100 cycles, the SSC-2M electrode surface exhibited numerous cracks (Figure 6g). In contrast, the SSC-5M electrode remained relatively intact, with almost no significant cracks observed after cycling (Figure 6h). However, due to the insufficient dispersion of Si NPs, the SSC-10M electrode showed a few cracks, even after 100 cycles (Figure 6i).

**Figure 7:**
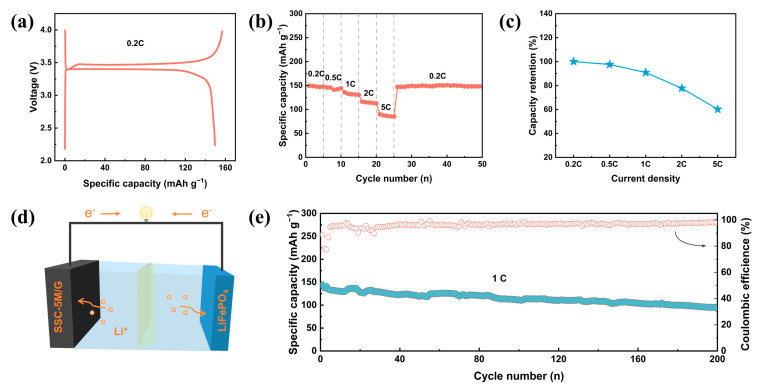
(**a**) Initial GCD curves at 0.2 C (1 C = 170 mAh g^−1^). (**b**,**c**) Rate performance and capacity retention. (**d**) The model of the full cell. (**e**) Long-term cycle performance of the SSC-5M/G//LiFePO_4_ full cell.

The practical applicability of the SSC-5M composite was assessed by systematically evaluating its full-cell performance. After the anode material was prelithiated, the SSC-5M/G//LiFePO_4_ full cell exhibited a discharge capacity of 149.55 mAh g^−1^ and a high ICE of 95.30% (Figure 7a). Notably, a reversible capacity of 87.75 mAh g^−1^ was achieved by the SSC-5M/G//LiFePO_4_ full cell, even at a high current density of 5 C (Figure 7b). As shown in Figure 7c, based on the capacity at 0.2 C, a capacity retention rate of 5 C was maintained at 65.48%. Furthermore, the SSC-5M/G//LiFePO_4_ full cell retained a specific capacity of 94.86 mAh g^−1^ after 200 cycles at 1 C, demonstrating excellent cycling stability (Figure 7e). These findings indicate that the SSC-5M composite material has significant potential for practical applications in energy storage devices.

## 4. Conclusions

In conclusion, this study employed spray freeze drying technology utilizing droplet phase separation in liquid nitrogen and the effect of ice crystal growth to successfully construct carbon nanostructures ranging from one-dimensional carbon nanowires to two-dimensional carbon nanosheets. This process achieved the uniform dispersion and effective encapsulation of Si NPs. Notably, SSC-5M demonstrated the following key advantages: (1) the folded ultrathin carbon nanosheets effectively mitigated the structural degradation resulting from continuous volume variations in Si; (2) the unique two-dimensional structure greatly improved Li^+^ diffusion and charge transfer; and (3) the overall conductivity of the material was improved by nitrogen-doped carbon structures. Consequently, SSC-5M achieved a reversible capacity of 1279 mAh g^−1^ after 100 cycles at 0.2 C and retained 677.1 mAh g^−1^ after 400 cycles at 1 C, presenting an innovative and practical approach for designing Si-based energy storage devices.

## Data Availability

Data underlying the results presented in this paper are not publicly available at this time but may be obtained from the authors upon reasonable request.

## Supplementary Materials

The following supporting information can be downloaded at https://www.mdpi.com/article/10.3390/nano15090661/s1, SEM; particle size distribution; fitted Raman spectra; XPS; N_2_ adsorption and desorption curves; TG curves; GCD curves; CV curves; Table of pore properties and electrochemical performance of SSCs and additional details on equations. Refs. [45,46,47,48,49,50,51] are cited in the Supplementary Materials.
