# Supplementary material for: Assembled Carbon Nanostructure Prepared by Spray Freeze Drying for Si-Based Anodes
Source: Nanomaterials (Basel). 2025 Apr 26;15(9):661. doi: 10.3390/nano15090661 (PMC12074266; doi:10.3390/nano15090661)
Supplement: Supplementary file 1 [file nanomaterials-15-00661-s001.zip › nanomaterials-3589164-supplementary.pdf]

Supplementary

Assembled Carbon Nanostructure Prepared by Spray Freeze Drying for Si-Based Anodes

Wanxiong Zhu [1], Liewen Guo [1], Kairan Li [1], Mengxue Shen [1], Chang Lu [1], Zipeng Jiang [2,3],

†Huaihe Song [1], Ang Li [1],*

[1] State Key Laboratory of Chemical Resources Engineering, Beijing Key Laboratory of Electrochemical Process and Technology for Materials, Beijing University of Chemical Technology, Beijing 100029, China

[2] Qinghai Provincial Key Laboratory of Advanced Materials and Applied Technology, Qinghai University, Xining, China

[3] College of Chemical Engineering, Qinghai University, Xining, China

* Correspondence：li_ang@buct.edu.cn

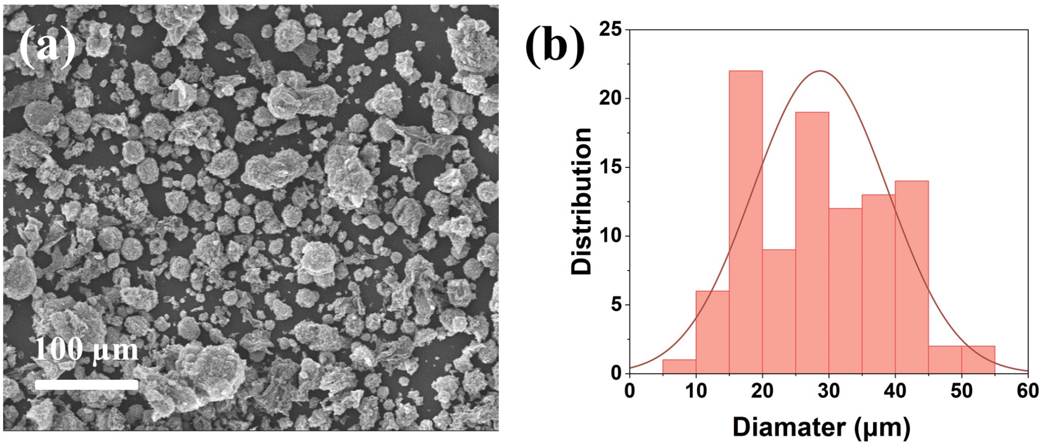

Figure S1. (a) Scanning electron microscope (SEM) images of SSC-5M and (b) its particle size distribution (PSD) graph.

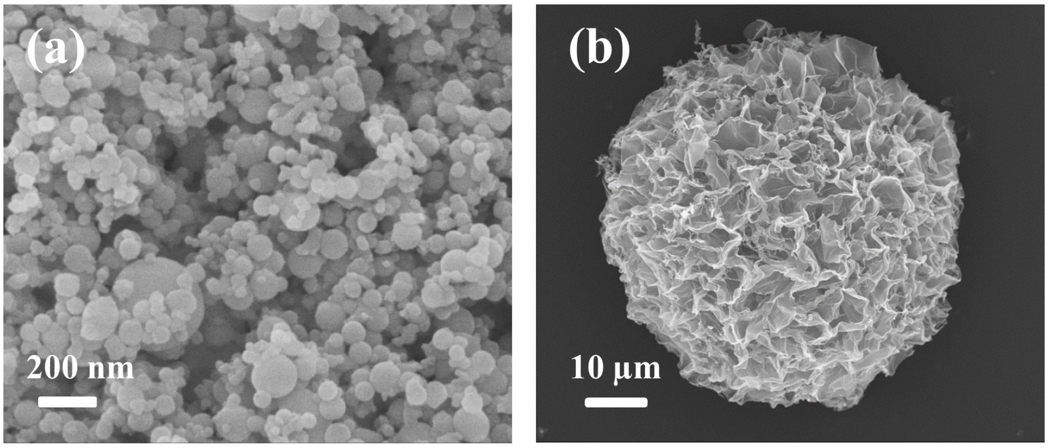

Figure S2. SEM images of (a) silicon nanoparticles, (b) Spherical assembly structure without incorporated silicon nanoparticles (CS-5M).

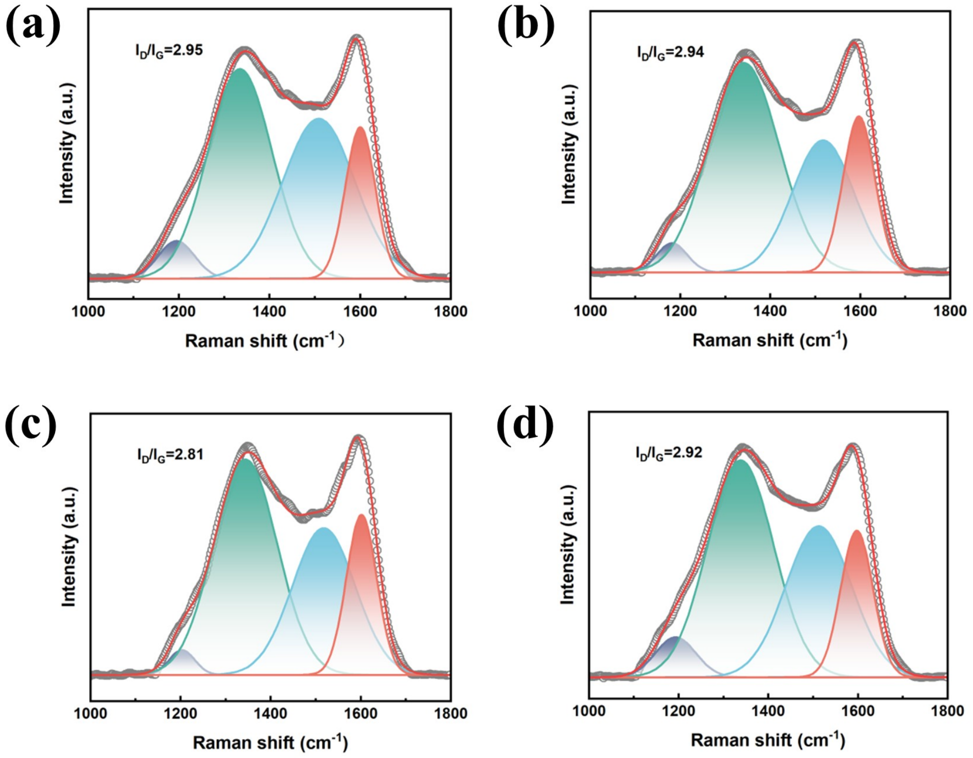

Figure S3. Fitted Raman spectra of (a) SSC-1M, (b) SSC-2M, (c) SSC-5M, (d) SSC-10M.

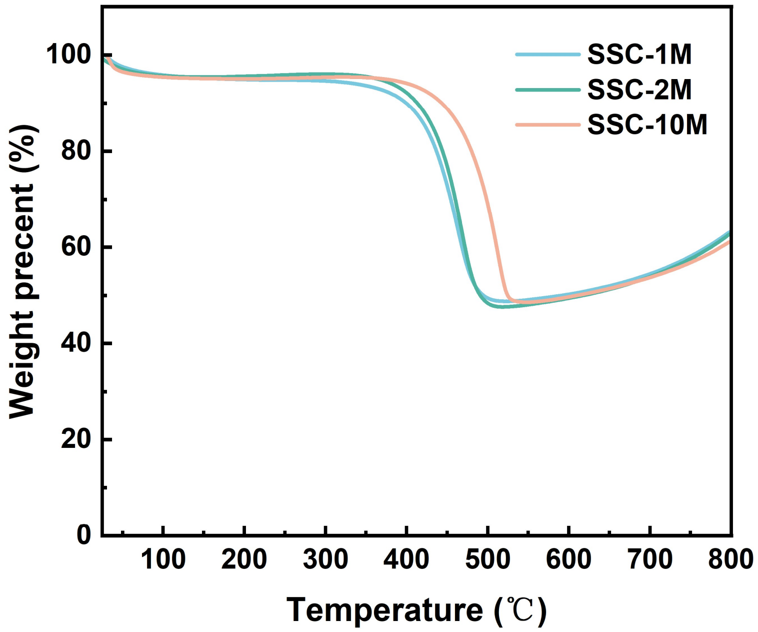

Figure S4. TGA curves of the other SSCs materials.

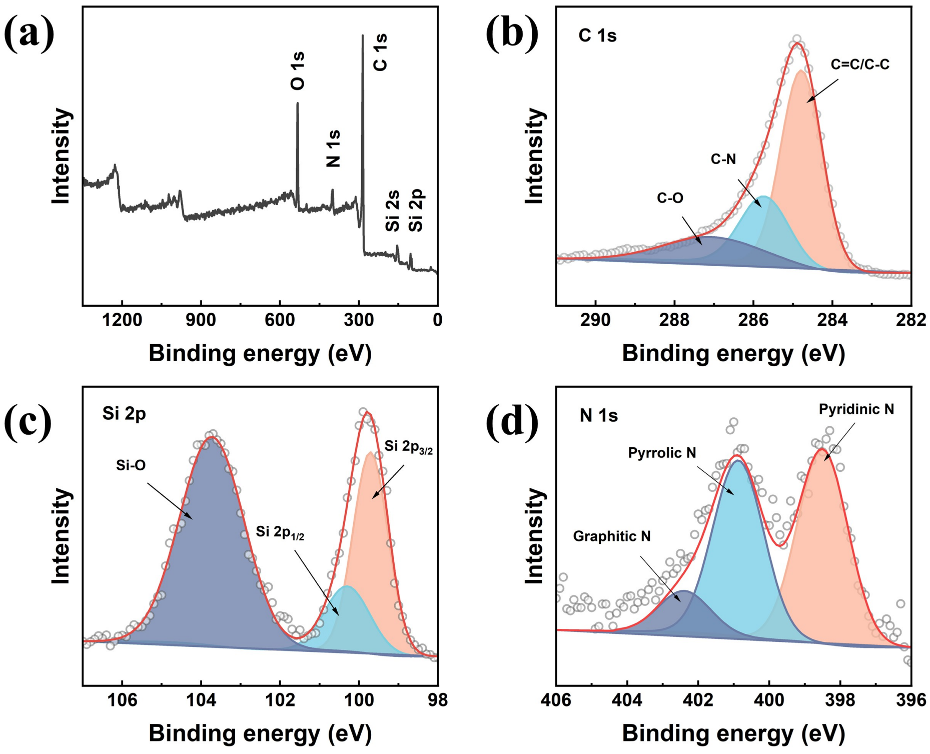

Figure S5. (a) XPS survey spectra of SSC-5M, and high-resolution XPS spectra of (b) C 1s, (c) Si 2p, (d) N 1s.

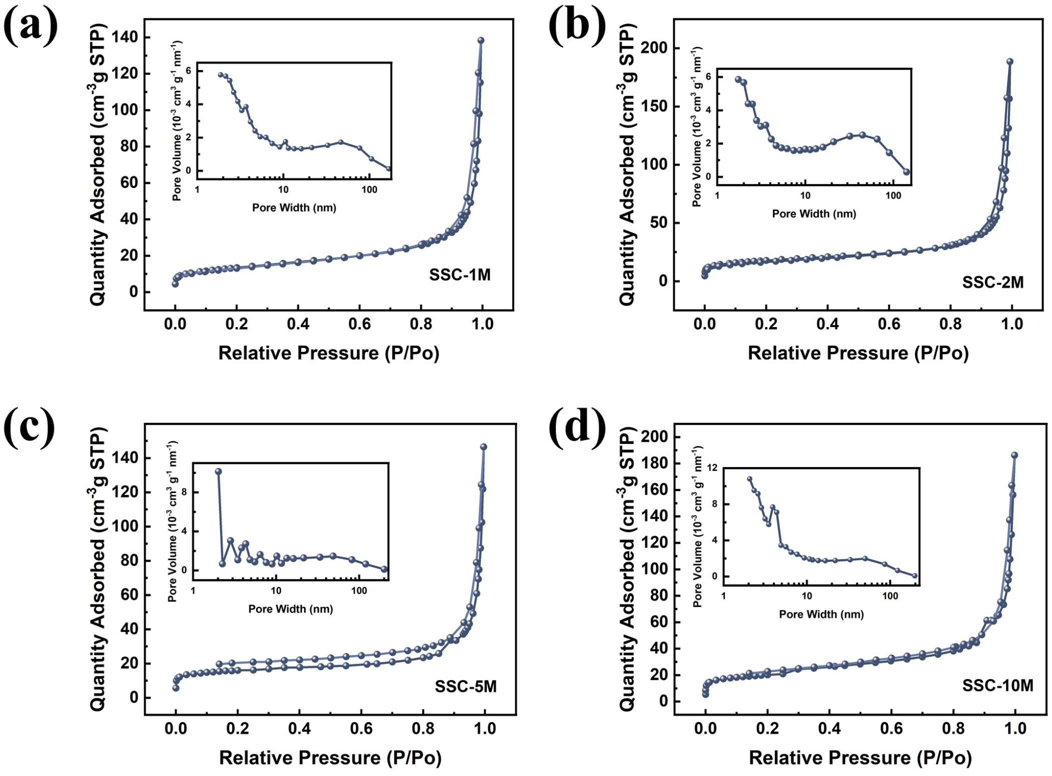

Figure S6. N_2 adsorption and desorption curves and pore size distribution curves (a) SSC-1M, (b) SSC-2M, (c) SSC-5M, (d) SSC-10M.

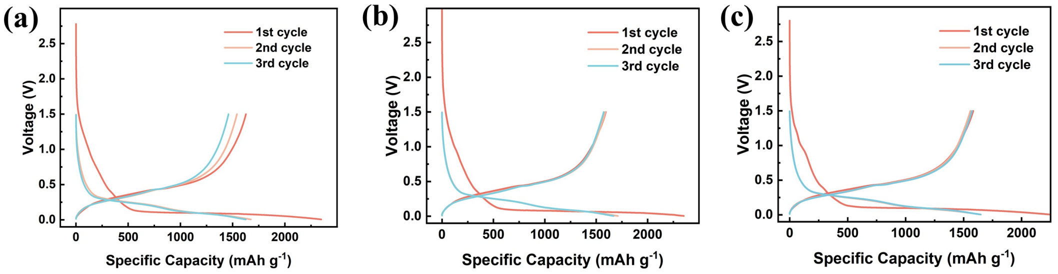

Figure S7. GCD curves for the first three cycles at 50mA g^{-1} current density (a) SSC-1M, (b) SSC-2M, and (c) SSC-10M.

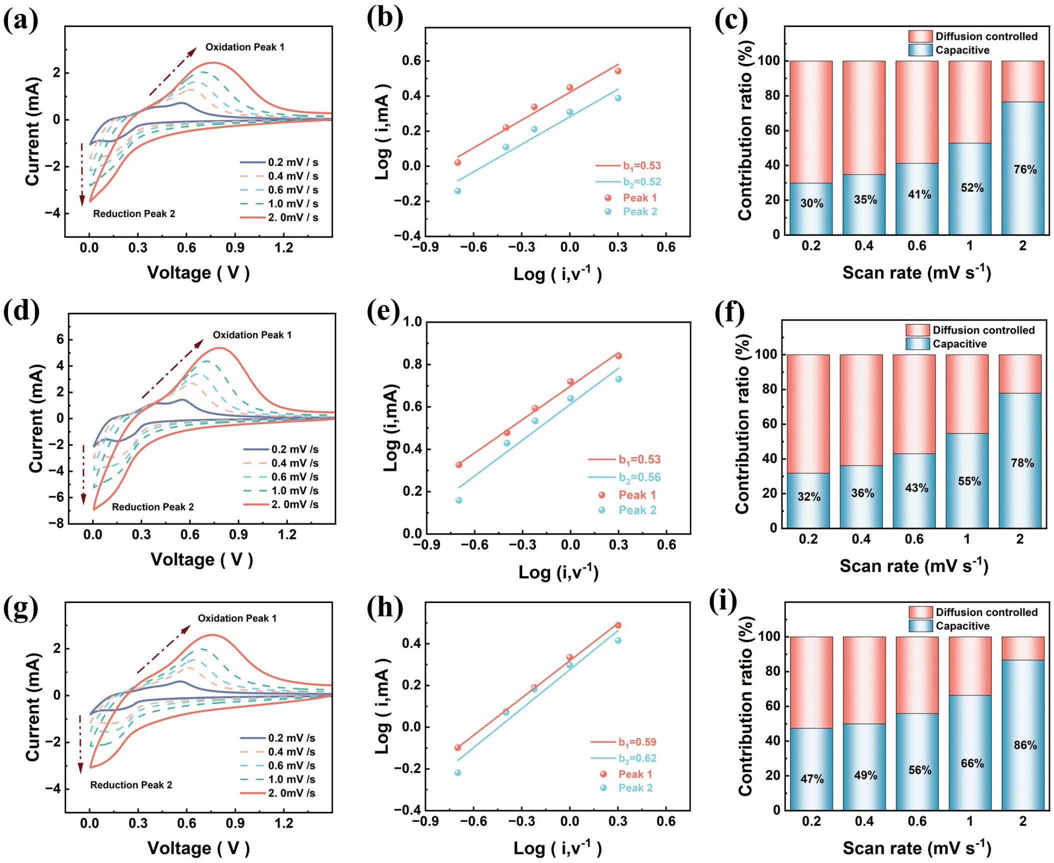

Figure S8. CV curves of SSC-1M, SSC-2M, SSC-10M at different scan rates (a, d, g); scan rate versus peak current (b, e, h); diffusion versus capacitance at different scan rates (c, f, i).

Supplementary Tables

Table S1 BET surface area and average pore diameter

Samples	SSC-1M	SSC-2M	SSC-5M	SSC-10M
Surface area ($m^2\ g^{-1}$)	46.44	50.68	59.68	72.23
Average pore width (nm)	21.29	25.47	17.81	15.94

Table S2 Electrochemical performance comparison with other materials

Method	ICE (%)	Silicon content (%)	Cycling performance ($mAh\ g^{-1}$)	Refs
ball milling (Gr@Si@C)	85.0	42.0	1167 (100cycles，0.5C)	[45]
Freeze drying (SiNC-3D)	70.1	65.5	1245 (100cycles，0.1A g^{-1})	[46]
Molten salt method (p-Si/CNT)	52.8	44.8	796.6 (100cycles,0.5A g^{-1})	[47]
CVD (PHC@Si@SC)	86.8	57.1	680 (70 cycles,0.2A g^{-1})	[48]
Electrospinning (PVA/Si/PAN)	71.7	~40	~900 (250cycles,0.5C)	[49]
Spray drying (PorousSi@C)	57.8	69.1	1170 (100cycles,0.2A g^{-1})	[50]
Spray drying (S-Si-C)	71.4	44.1	1055 (100cycles,0.5A g^{-1})	[51]
**This work**	**74.94**	**49.2**	**1279 (100cycles,0.2C)**	

Supplementary Equations

Equation S1

$$\Delta \mathrm{T} = \frac{2\sigma V r T_M}{\Delta H}$$

where σ is the surface tension of the liquid, V the droplet volume, r the curvature, T_M the melting temperature, and ΔH the latent heat of phase transition.

Equation S2

$$\mathrm{I} = \mathrm{Aexp}\left(-\frac{\Delta G^* + E_D}{kT}\right)$$

where A is a constant, positively correlated with I, which can represent the polymer embryo concentration, ΔG^* is the activation energy required to transfer the polymer embryo from the parent phase to the new phase, E_D is the diffusion activation energy, and k is Boltzmann's constant.

Equation S3

$$\mathrm{D_{Li^+}} = \frac{4}{\pi\tau}\left(\frac{\mathrm{m_B V_B}}{\mathrm{M_B S}}\right)^2 \left(\frac{\Delta \mathrm{E_s}}{\Delta \mathrm{E_\tau}}\right)$$

In the above equations, τ, $\mathrm{m_B}$, and $\mathrm{V_B}$ represent the relaxation time, mass and molar volume of the electrode active material, respectively, while $\mathrm{M_B}$ and S denote the molar mass of the active material and the contact area of the electrode with the electrolyte, respectively. $\Delta \mathrm{E_s}$ is the amount of the voltage change in the steady state phase, whereas $\Delta \mathrm{E_\tau}$ is the amount of the voltage change after removing the IR drop in the constant-current charging and discharging process.

Equation S4

$$\mathrm{i} = \mathrm{a}v^{b}$$

$$\mathrm{Log}(i) = bLog(v) + Log(a)$$

In the above equation, i and v are the peak current and scan rate, respectively, and a and b are variable parameters. Generally, the value of b can be obtained by calculating the slope of the $\mathrm{Log}(i)$- $Log(v)$ curve, which is used to indicate the storage mechanism of lithium in the electrode material. When b = 0.5, it indicates that the electrode material is mainly controlled by diffusion process, and when b = 1, the electrode material is controlled by capacitive process.

Equation S5

$$\mathrm{i(V)} = \mathrm{k_1 v} + \mathrm{k_2 v^{1/2}}$$

In addition, according to the above equation, the total current consists of two parts, k_1v and $k_2v^{1/2}$, where k_1v represents the capacitor-controlled part and $k_2v^{1/2}$ represents the diffusion-controlled part.

References

45 Kim, M. J.; Lee, I.; Lee, J. W.; Yoon, D.; Kim, J. H.; Lee, S.; Kim, K.; Kim, P. J.; Choi, J.; Kang, Y. C.; Jung, D. S., A Novel Structured Si-Based Composite with 2D Structured Graphite for High-Performance Lithium-Ion Batteries. *Small* **2024,** *20*, 2405005.

46 Fan, P.; Lou, S.; Sun, B.; Wu, L.; Qian, Z.; Mu, T.; Ma, Y.; Cheng, X.; Gao, Y.; Zuo, P.; Du, C.; Yin, G., Improving electrochemical performance of Nano-Si/N-doped carbon through tunning the microstructure from two dimensions to three dimensions. *Electrochimica Acta* **2020,** *332*, 135507.

47 Zhang, Q.; Xi, B.; Chen, W.; Feng, J.; Qian, Y.; Xiong, S., Synthesis of carbon nanotubes-supported porous silicon microparticles in low-temperature molten salt for high-performance Li-ion battery anodes. Nano Research 2022, 15 (7), 6184-6191.

48 Lv, D.; Yang, L.; Song, R.; Yuan, H.; Luan, J.; Liu, J.; Hu, W.; Zhong, C., A hierarchical porous hard carbon@Si@soft carbon material for advanced lithium-ion batteries. Journal of Colloid and Interface Science 2025, 678, 336-342.

49 Pei, Y.; Wang, Y.; Chang, A.-Y.; Liao, Y.; Zhang, S.; Wen, X.; Wang, S., Nanofiber-in-microfiber carbon/silicon composite anode with high silicon content for lithium-ion batteries. Carbon 2023, 203, 436-444.

50 Wang, D.; Kong, L.; Zhang, F.; Liu, A.; Huang, H.; Liu, Y.; Shi, Z., Porous carbon-coated silicon composites for high performance lithium-ion batterie anode. *Applied Surface Science* **2024,** *661*, 160076.

51 Qiao, Y.; Hu, Y.; Qian, Z.; Qu, M.; Liu, Z., An innovative strategy for constructing multicore yolk-shell Si/C anodes for lithium-ion batteries. Journal of Colloid and Interface Science 2025, 684, 678-689.
